# Combined red blood cell distribution width and neutrophil-to-lymphocyte ratio and risk of composite cardiovascular events in patients with moderate-to-severe obstructive sleep apnea: a prospective cohort study

**DOI:** 10.3389/fmed.2026.1799058

**Published:** 2026-03-11

**Authors:** Ningzhen Li, Hongtao Liu, Linlin Zhang, Mingjing Chang, Yuhong Xia

**Affiliations:** Respiratory and Critical Care Medicine Ward 2, Xinxiang Central Hospital, Xinxiang, Henan, China

**Keywords:** cardiovascular events, neutrophil-to-lymphocyte ratio, obstructive sleep apnea, predictive value, red blood cell distribution width

## Abstract

**Objective:**

To evaluate the prognostic association of baseline red blood cell distribution width (RDW), neutrophil-to-lymphocyte ratio (NLR), and their combined index with 3-year composite cardiovascular events in moderate-to-severe obstructive sleep apnea (OSA) patients.

**Methods:**

This prospective cohort study enrolled 620 patients with moderate-to-severe OSA (AHI ≥ 15 events/h) from 2020–2022. Participants were categorized by baseline medians into low-risk (both low), intermediate-risk (one high), and high-risk (both high) groups. The primary outcome was composite cardiovascular events. Cox regression, ROC curves, NRI, and IDI assessed predictive performance.

**Results:**

During median follow-up of 36.2 months, 72 events occurred (11.6% rate). Each 1% increase in RDW (HR = 1.32, 95% CI: 1.15–1.52) and each 1-unit increase in NLR (HR = 1.41, 95% CI: 1.20–1.66) were independently associated with cardiovascular events (both *p* < 0.001). The high-risk group showed 2.98-fold higher risk than low-risk group (HR = 2.98, 95%CI: 1.36–6.52, *p* = 0.006) with significant dose–response relationship (*p* = 0.002). The combined RDW-NLR index showed better discrimination (AUC = 0.73, 95%CI: 0.65–0.81) compared with RDW alone (0.65) or NLR alone (0.67). Adding the combined index to traditional risk factors and OSA parameters significantly improved reclassification (NRI = 0.42, *p* = 0.001; IDI = 0.032, *p* = 0.009). The association was more pronounced in patients aged ≥60 years, males, and those with poor CPAP adherence.

**Conclusion:**

The combined RDW-NLR index is a simple, accessible prognostic marker of cardiovascular events in moderate-to-severe OSA patients, providing additional risk stratification value beyond traditional risk factors for clinical risk stratification.

## 1 Introduction

Obstructive sleep apnea (OSA) is characterized by recurrent upper-airway collapse during sleep, leading to intermittent hypoxia, sympathetic surges and sleep fragmentation (1). Recent estimates indicate that 936 million adults aged 30–69 years have OSA, including 425 million with moderate-to-severe disease (2, 3). Beyond daytime sleepiness and impaired quality of life, OSA is now recognized as an independent cardiometabolic risk factor. Meta-analyses report 1.5–2.5-fold higher risks of fatal or non-fatal cardiovascular events, stroke and sudden death in moderate-to-severe cases compared with matched community controls (4, 5). Consequently, early identification of high-risk individuals remains a public health priority.

Contemporary risk algorithms do not incorporate disease-specific mechanisms of OSA and consequently underestimate event probability in this population (6). Intermittent hypoxia triggers systemic inflammation, oxidative stress, endothelial dysfunction and thrombophilia—pathways poorly captured by traditional covariates such as age, hypertension or diabetes (7). Surrogate biomarkers that are inexpensive, reproducible and reflective of these mechanisms could therefore refine bedside risk stratification and guide targeted intervention.

The neutrophil-to-lymphocyte ratio (NLR) and red cell distribution width (RDW) are readily available from routine complete blood counts without additional cost. NLR integrates neutrophil-driven innate immunity and lymphocyte-mediated adaptive immunity, and has demonstrated utility for risk stratification in moderate-to-severe OSA (8). Moreover, NLR levels correlate positively with the apnea–hypopnea index (AHI), a key metric for quantifying OSA severity (9). RDW quantifies erythrocyte size heterogeneity, a surrogate of ineffective erythropoiesis and inflammatory bone-marrow suppression (10). RDW elevation has been recognized as a significant prognostic marker for in-hospital mortality in patients with acute myocardial infarction (11). Although intermittent hypoxia in OSA is closely linked to increased RDW, and RDW is positively correlated with the apnea-hypopnea index, prospective studies exploring the specific link between OSA-related RDW elevation and long-term cardiovascular outcomes remain insufficient (12).

Although NLR reflects systemic inflammation and RDW reflects hypoxic stress, their combined utility in risk assessment remains unexplored (13). Retrospective analyses in surgical cohorts have confirmed the additive value of such a combined approach, yet corresponding evidence in obstructive sleep apnea (OSA) is lacking (14). Notably, prior research has seldom adjusted for polysomnographic severity indices (e.g., nocturnal desaturation indices) or traditional cardiovascular confounders, creating a knowledge gap that limits the clinical application of these low-cost hematological markers (15).

Therefore, we conducted a prospective cohort study of treatment-naive moderate-to-severe OSA patients without baseline cardiovascular disease. We examined whether baseline NLR, RDW or an integrated RDW-NLR score is associated with 3-year composite cardiovascular events, assessed whether it provides additional prognostic information beyond conventional and OSA-specific risk factors, and examined dose–response relationships. We hypothesized that the combined index would outperform single markers and provide a simple, universally applicable bedside tool for cardiovascular risk stratification in OSA.

## 2 Methods

### 2.1 Study design

A single-center prospective cohort study was performed, with participants recruited from the Sleep Center of our hospital between January 2020 and December 2022. The study protocol was approved by the Institutional Review Board of our hospital (Approval No: 2021-036-01[K]), and all participants provided written informed consent prior to enrollment, in compliance with the ethical principles of the Declaration of Helsinki.

### 2.2 Study population

Eligible participants were aged 18–75 years, diagnosed with moderate-to-severe OSA by polysomnography according to the American Academy of Sleep Medicine (AASM) scoring criteria. Moderate-to-severe OSA was defined as an apnea-hypopnea index (AHI) ≥ 15 events/h, with hypopneas scored as a ≥ 30% reduction in nasal airflow accompanied by either a ≥ 3% oxygen desaturation or an electroencephalographic arousal (16). All participants were clinically stable at enrollment, defined as: (i) no acute illness (infection, fever, acute exacerbation of chronic disease) within 2 weeks prior to enrollment; (ii) no hospitalization or emergency department visit within 1 month prior to enrollment; (iii) no acute inflammatory conditions (C-reactive protein <10 mg/L); and (iv) hematocrit and hemoglobin within stable ranges without acute bleeding. Patients with acute conditions affecting CBC parameters were excluded to ensure biomarker stability. All participants had complete baseline blood routine data available, including RDW, neutrophil count, and lymphocyte count, and voluntarily agreed to complete long-term follow-up. Exclusion criteria included a previous diagnosis of cardiovascular diseases (such as myocardial infarction, stroke, heart failure, or coronary revascularization), hematological diseases, autoimmune diseases, malignant tumors, chronic infectious diseases, acute inflammation, surgical history, severe trauma, or systemic corticosteroid/immunosuppressive therapy within 1 month prior to enrollment, severe liver or renal insufficiency, thyroid dysfunction, continuous positive airway pressure (CPAP) treatment adherence ≥4 h/night (excluding those who initiated CPAP during follow-up), incomplete clinical data, or inability to cooperate with follow-up. Sample size was estimated using PASS 15.0 software based on previous similar studies, with effect sizes set as hazard ratios (HR) of 1.75 for RDW and 1.85 for NLR regarding composite cardiovascular events; a two-sided *α* of 0.05, power of 90%, expected 3-year event rate of 10.3%, proportion of high-risk group of 50% (median-stratified balanced grouping), and expected loss to follow-up rate of 15% were used, resulting in a minimum required sample size of 320 cases.

### 2.3 Data collection

Baseline demographic characteristics (age, gender, ethnicity) and lifestyle factors (smoking history, drinking history, exercise habits) were collected via standardized questionnaires (Supplementary material 1), while height, weight, and blood pressure (average of 3 measurements) were measured to calculate body mass index (BMI); clinical history (hypertension, diabetes, dyslipidemia) and medication use were extracted from electronic medical records. All participants underwent overnight polysomnography monitoring for ≥7 h. Fasting venous blood (5 mL) was collected in the morning immediately after polysomnography completion (between 6:00 and 8:00 a.m.) after ≥8 h of overnight fasting, and RDW (expressed as RDW-CV), neutrophil count, and lymphocyte count were measured using an automatic hematology analyzer within 2 h to avoid hemolysis interference, with NLR calculated as neutrophil count divided by lymphocyte count. Polysomnography results were independently analyzed by two senior sleep physicians, with discrepancies resolved by consensus.

### 2.4 Exposure definition and grouping

RDW and NLR were analyzed both as continuous variables and categorized into quartiles (Q1–Q4) with Q1 as the reference group. A combined risk index was constructed by cross-stratification: RDW and NLR were divided into high (≥median) and low (<median) subgroups based on baseline medians, resulting in three risk groups: low-risk (low RDW + low NLR), intermediate-risk (single high index), and high-risk (high RDW + high NLR).

### 2.5 Outcome measures and follow-up

The primary outcome was first-ever composite cardiovascular events, comprising five prespecified components: (i) cardiovascular death (sudden cardiac death, fatal myocardial infarction, fatal stroke, or death from other cardiovascular causes); (ii) non-fatal myocardial infarction (Third Universal Definition, 2012); (iii) non-fatal ischemic stroke (TOAST classification, confirmed by CT/MRI, with transient ischemic attack excluded); (iv) unplanned hospitalization for acute heart failure (primary diagnosis requiring intravenous diuretic or inotropic therapy); and (v) urgent or emergent coronary revascularization (percutaneous coronary intervention or coronary artery bypass grafting performed for acute coronary syndromes). Elective revascularization procedures for stable coronary artery disease were excluded.

#### 2.5.1 Event ascertainment pathway

Participants were followed up for 3 years via scheduled outpatient visits and telephone interviews at 6 months, 1 year, 2 years, and 3 years. Suspected events were identified through multiple sources: (a) direct participant report during follow-up contacts; (b) review of electronic medical records from our hospital and linked regional healthcare database; (c) discharge summaries and imaging reports from external hospitals; and (d) death certificates from the National Mortality Surveillance System. For participants lost to routine follow-up, supplementary information was obtained via family interviews or the national medical insurance claims database. The last follow-up time was defined as the date of the last effective contact, with the loss-to-follow-up rate controlled below 15%.

#### 2.5.2 Endpoint adjudication

All suspected cardiovascular events were independently reviewed by a blinded endpoint adjudication committee comprising two cardiologists and one neurologist. Committee members were blinded to baseline RDW, NLR, and all other biomarker data to prevent ascertainment bias. Each event was classified using standardized definitions with source documentation (hospital records, ECGs, troponin values, CT/MRI reports, angiography images, death certificates). Discrepancies were resolved by consensus or consultation with a third senior specialist. Only events occurring after enrollment and within the 3-year follow-up window were counted.

#### 2.5.3 Handling of multiple events

The analysis used a time-to-first-event approach. Only the first qualifying component event for each participant was included in the primary composite endpoint; subsequent recurrent events (e.g., second MI or repeat revascularization) were not counted as additional outcomes. Participants were censored at the time of their first event for the primary analysis. For secondary analyses examining individual components, competing risk models were applied with non-cardiovascular death as a competing event.

### 2.6 Statistical analysis

Missing data were assessed for all variables. The proportion of missing data for key variables was minimal: RDW < 1%, NLR < 1%, AHI < 1%, SLT90 < 1%, minimum SpO₂ < 1%, BMI < 1%, age <1%, and traditional risk factors <1%. Given the low missingness rate (<5%), complete-case analysis was employed for the primary analysis. CPAP adherence data were available for 360 participants (58.1%); analyses stratified by CPAP adherence were conducted in this subset only. All analyses were performed using R version 4.2.0. A two-sided *p* < 0.05 was considered statistically significant. Continuous variables are presented as mean ± standard deviation or median (interquartile range), and compared using the independent *t*-test or Wilcoxon rank-sum test. Categorical variables are expressed as frequencies (percentages) and compared using the χ^2^ test. Cox proportional hazards regression was used to assess associations between exposure variables and composite cardiovascular events, with the proportional hazards assumption evaluated using Schoenfeld residuals (all *p* > 0.05). For the primary composite endpoint, we employed a time-to-first-event analysis. Participants were censored at the earliest of: (i) first qualifying cardiovascular event; (ii) death from non-cardiovascular causes; (iii) loss to follow-up; or (iv) study completion at 3 years. Recurrent events were not counted. We did not use logistic regression for a fixed horizon. All participants had variable follow-up (median 36.2 months, IQR 34.5–37.8 months); the 3-year cumulative event rate (11.6%) was estimated using the Kaplan–Meier method. Candidate confounders were screened via univariate Cox regression (*p* < 0.10). Age, sex, and BMI were forced into all models. Three models were constructed: Model 1 (unadjusted), Model 2 (adjusted for traditional risk factors: age, sex, BMI, smoking, alcohol, hypertension, diabetes, dyslipidemia), and Model 3 (fully adjusted, plus AHI, SLT90, minimum SpO₂). Model discrimination was evaluated using ROC curves (AUC), C-index, and AIC. NRI and IDI compared the combined RDW-NLR index with individual markers. Stratified analyses were conducted by age, sex, and CPAP adherence. A sensitivity analysis excluded participants with follow-up <1 year.

## 3 Results

### 3.1 Study population and baseline characteristics

**Figure 1:**
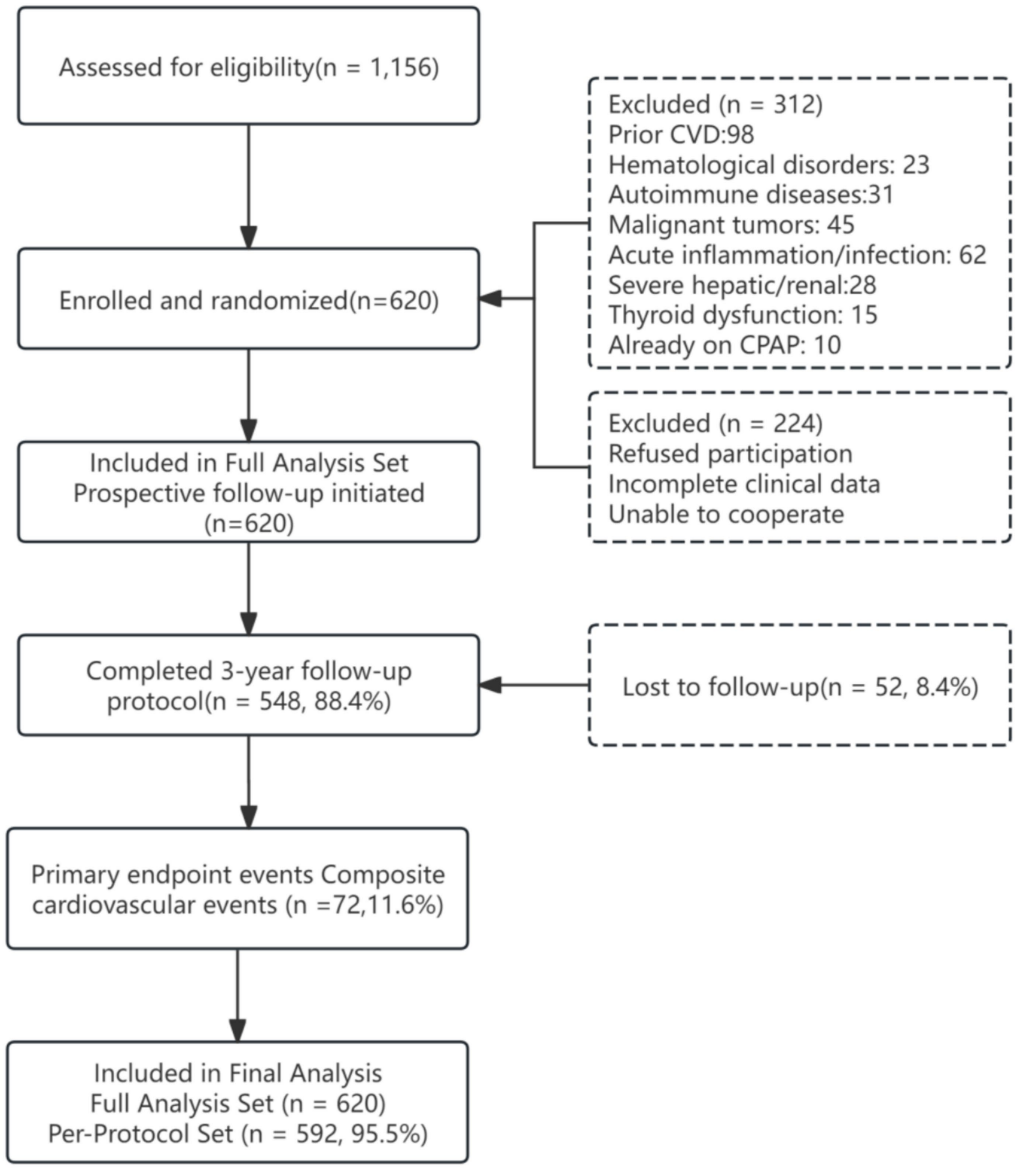
Patient screening and enrollment process.

Between January 2020 and December 2022, a total of 1,156 consecutive patients with suspected moderate-to-severe OSA were assessed for eligibility at the Sleep Center. Following initial screening, 312 patients were excluded for not meeting inclusion criteria (including: prior cardiovascular disease *n* = 98, hematological disorders *n* = 23, autoimmune diseases *n* = 31, malignant tumors *n* = 45, acute inflammation or infection *n* = 62, severe hepatic or renal insufficiency *n* = 28, thyroid dysfunction *n* = 15, and CPAP treatment ≥4 h/night *n* = 10). An additional 224 patients were excluded due to refusal to participate, incomplete clinical data, or inability to cooperate with long-term follow-up (Figure 1). A total of 620 eligible participants were enrolled in this study, with a median follow-up duration of 36.2 months (interquartile range: 34.5–37.8 months). During the follow-up period, 72 composite cardiovascular events occurred, resulting in an overall 3-year event rate of 11.6% (72/620), which was consistent with the expected event rate in the sample size calculation. Table 1 presents baseline characteristics by event status and risk group. Participants who experienced cardiovascular events were significantly older (median age: 62.3 vs. 54.7 years, *p* < 0.001) and had a higher BMI (29.8 ± 3.5 vs. 27.6 ± 3.2 kg/m^2^, *p* = 0.003) compared with those without events. The proportion of participants with hypertension (68.6% vs. 42.1%, *p* < 0.001), diabetes mellitus (45.7% vs. 23.3%, *p* = 0.002), and dyslipidemia (57.1% vs. 36.5%, *p* = 0.006) was significantly higher in the event group. Regarding OSA-related parameters, the event group had a higher AHI (34.2 ± 10.5 vs. 28.7 ± 9.8 events/h, *p* = 0.004), longer SLT90 (18.5 ± 8.2% vs. 12.3 ± 7.6%, *p* < 0.001), and lower minimum SpO₂ (78.3 ± 6.5% vs. 83.5 ± 5.8%, *p* < 0.001). Laboratory indicators showed that the event group had significantly higher baseline RDW (13.8 ± 1.2% vs. 12.6 ± 1.0%, *p* < 0.001) and NLR (2.8 ± 0.9 vs. 2.1 ± 0.7, *p* < 0.001) compared with the non-event group. There were no significant differences in gender distribution, smoking history, drinking history, or medication use between the two groups (all *p* > 0.05). When stratified by the combined RDW-NLR risk groups, 197 participants (31.8%) were classified into the low-risk group, 325 (52.4%) into the intermediate-risk group, and 98 (15.8%) into the high-risk group. The high-risk group had the highest proportion of elderly participants, comorbidities, and severe OSA indicators compared with the other two groups (all *p* < 0.05).

**Table 1:** Baseline characteristics of the study population.

Variables	Total (*n* = 620)	No Event (*n* = 548)	Event (*n* = 72)	*p* value	Low-risk (*n* = 197)	Intermediate-risk (*n* = 325)	High-risk (*n* = 98)	*p* value
Demographic characteristics								
Age, years	55.8 ± 9.2	54.7 ± 8.9	62.3 ± 8.5	<0.001	52.1 ± 8.3	56.4 ± 9.1	61.7 ± 8.7	<0.001
Male, *n* (%)	418 (67.4)	370 (67.5)	48 (66.7)	0.325	135 (68.5)	217 (66.8)	66 (67.3)	0.931
BMI, kg/m^2^	28.1 ± 3.3	27.6 ± 3.2	29.8 ± 3.5	0.003	26.8 ± 2.9	28.3 ± 3.2	30.5 ± 3.6	<0.001
Lifestyle factors								
Smoking history, *n* (%)	190 (30.6)	167 (30.5)	23 (31.9)	0.892	60 (30.5)	101 (31.1)	29 (29.6)	0.984
Drinking history, *n* (%)	161 (26.0)	142 (25.9)	19 (26.4)	0.978	50 (25.4)	85 (26.2)	26 (26.5)	0.991
Comorbidities								
Hypertension, *n* (%)	283 (45.6)	230 (42.0)	53 (73.6)	<0.001	53 (27.3)	161 (49.5)	69 (70.4)	<0.001
Diabetes mellitus, *n* (%)	159 (25.6)	126 (23.0)	33 (45.8)	0.002	31 (15.7)	91 (28.0)	37 (37.8)	0.003
Dyslipidemia, *n* (%)	229 (36.9)	191 (34.9)	38 (52.8)	0.006	48 (24.4)	126 (38.8)	55 (56.1)	<0.001
OSA-related parameters								
AHI, events/h	29.8 ± 10.1	28.7 ± 9.8	34.2 ± 10.5	0.004	26.3 ± 9.2	30.1 ± 10.0	35.7 ± 10.8	<0.001
SLT90, %	13.2 ± 7.8	12.3 ± 7.6	18.5 ± 8.2	<0.001	9.8 ± 6.5	13.5 ± 7.7	21.6 ± 8.5	<0.001
Minimum SpO₂, %	82.6 ± 6.1	83.5 ± 5.8	78.3 ± 6.5	<0.001	85.2 ± 5.3	82.4 ± 6.0	77.1 ± 6.8	<0.001
Laboratory indicators								
RDW-CV, %	12.8 ± 1.1	12.6 ± 1.0	13.8 ± 1.2	<0.001	11.9 ± 0.8	12.8 ± 0.9	14.2 ± 1.1	<0.001
NLR	2.2 ± 0.8	2.1 ± 0.7	2.8 ± 0.9	<0.001	1.8 ± 0.6	2.2 ± 0.7	3.1 ± 0.9	<0.001

Data are presented as mean ± SD (continuous variables) or *n* (%) (categorical variables). Missing data were <1% for all variables shown; complete-case analysis includes all 620 participants. Comparisons were performed using *t*-test/Wilcoxon rank-sum test (continuous variables) or χ^2^ test (categorical variables). BMI, body mass index; AHI, apnea-hypopnea index; SLT90, percentage of time with SpO₂ < 90%; SpO₂, oxygen saturation; RDW-CV, red blood cell distribution width-coefficient of variation; NLR, neutrophil-to-lymphocyte ratio.

### 3.2 Correlation between RDW and NLR

**Figure 2:**
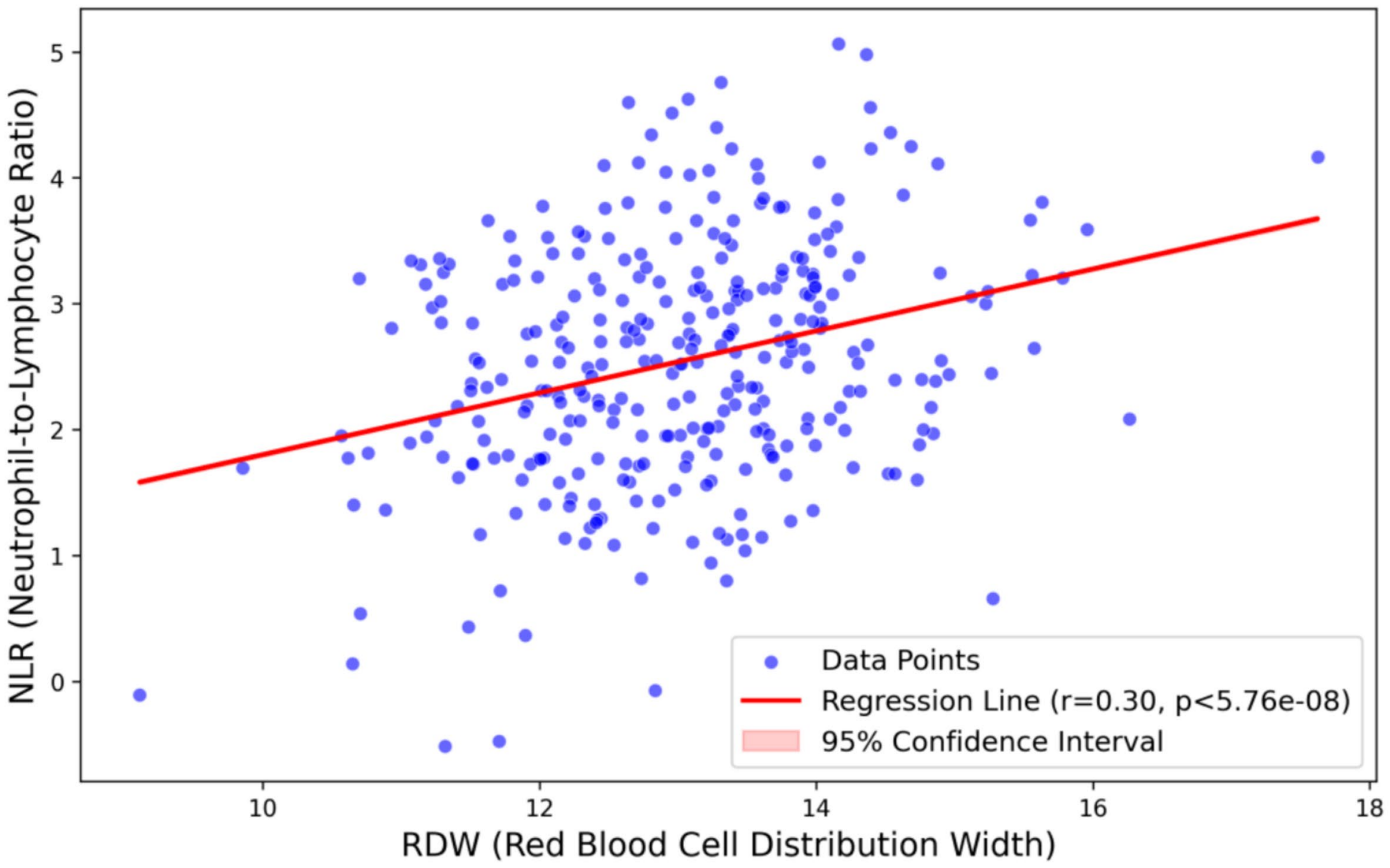
Correlation scatter plot of RDW and NLR. Spearman correlation analysis shows a moderate positive correlation between RDW and NLR (*r* = 0.230, *p* < 0.001). The solid line represents the linear regression fit, and the shaded area represents the 95% confidence interval. RDW, red blood cell distribution width; NLR, neutrophil-to-lymphocyte ratio.

Spearman correlation analysis showed that baseline RDW was positively correlated with NLR in the overall study population (*r* = 0.30, *p* < 0.001). Figure 2 illustrates this moderate positive correlation, suggesting a potential synergistic relationship between systemic inflammation (reflected by NLR) and erythrocyte heterogeneity (reflected by RDW) in patients with moderate-to-severe OSA.

### 3.3 Association between RDW, NLR, and composite cardiovascular events

Univariate Cox regression analysis showed that higher RDW and NLR were significantly associated with increased risk of composite cardiovascular events. When analyzed as continuous variables, each 1% increase in RDW was associated with a 32% higher risk (HR = 1.32, 95%CI: 1.15–1.52, *p* < 0.001), and each 1-unit increase in NLR was associated with a 41% higher risk (HR = 1.41, 95%CI: 1.20–1.66, *p* < 0.001).

When categorized by quartiles, compared with Q1, Q4 of RDW was associated with a 2.31-fold increased risk (HR = 2.31, 95%CI: 1.08–4.95, *p* = 0.032) in the unadjusted model, which remained marginally significant after full adjustment (HR = 2.12, 95%CI: 0.99–4.54, *p* = 0.053). For NLR, Q4 vs. Q1 was associated with a 2.58-fold higher risk in the unadjusted model (HR = 2.58, 95%CI: 1.21–5.49, *p* = 0.014), which remained significant after full adjustment (HR = 2.35, 95%CI: 1.08–5.12, *p* = 0.032).

**Figure 3:**
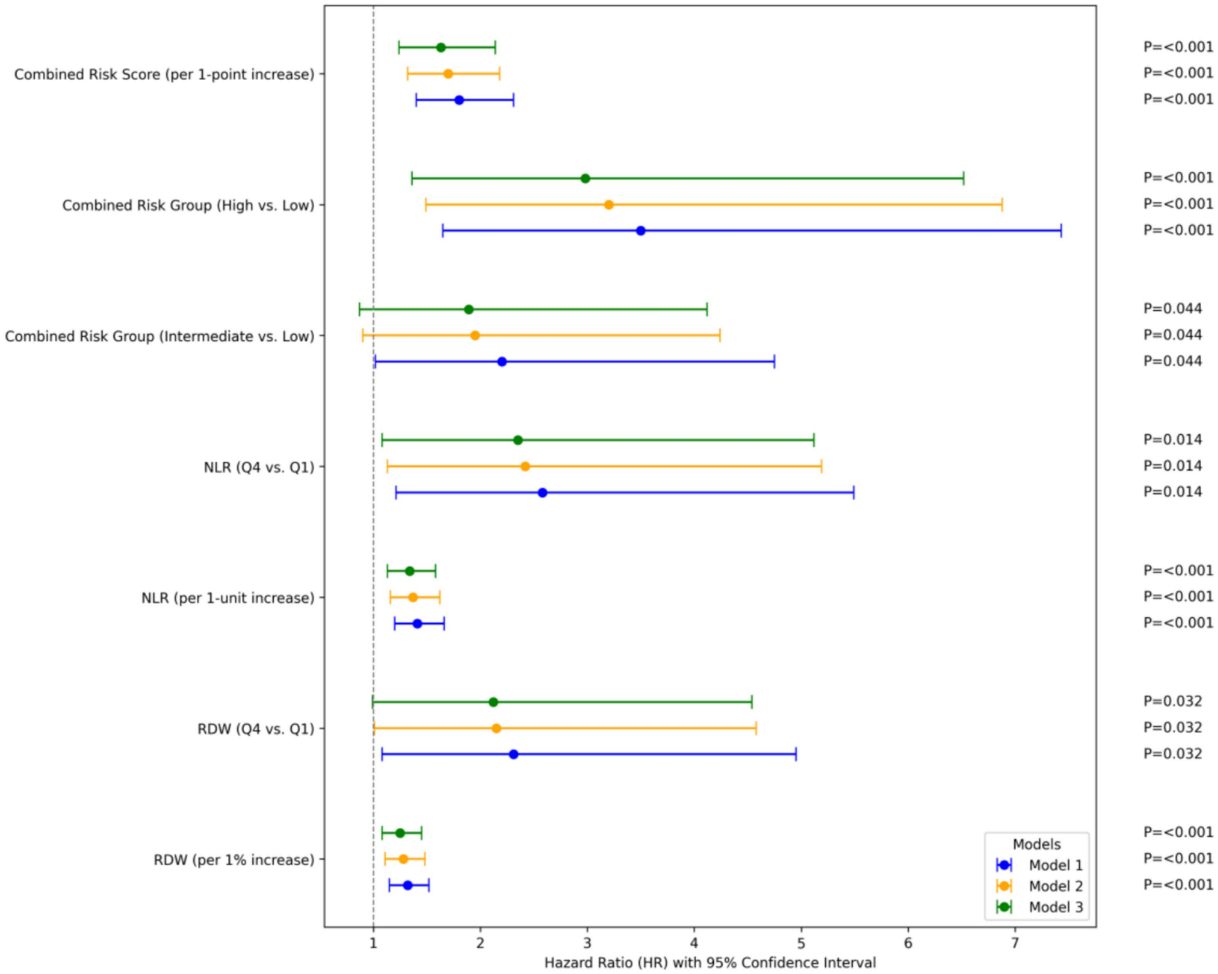
Cox proportional hazards regression results of RDW, NLR, and combined indices for composite cardiovascular events. RDW, red blood cell distribution width; NLR, neutrophil-to-lymphocyte ratio; HR, hazard ratio; CI, confidence interval. Traditional risk factors in Model 2 include age, gender, body mass index (BMI), smoking history, drinking history, hypertension, diabetes mellitus, and dyslipidemia. Model 3 further adjusts for obstructive sleep apnea (OSA)-related indicators (apnea-hypopnea index [AHI], percentage of time with oxygen saturation <90% [SLT90], minimum oxygen saturation [SpO_2_]).

Compared with the low-risk group, the intermediate-risk group had a non-significant increased risk (HR = 1.89, 95%CI: 0.87–4.12, *p* = 0.108) in the fully adjusted model, while the high-risk group had a 2.98-fold higher risk (HR = 2.98, 95%CI: 1.36–6.52, *p* = 0.006). The risk of events showed a significant dose–response relationship across the three risk groups (*P* for trend = 0.002 in Model 3). As a continuous variable, the combined risk score (0 = low risk, 1 = intermediate risk, 2 = high risk) was independently associated with composite cardiovascular events (HR = 1.63, 95%CI: 1.24–2.14, *p* < 0.001 in Model 3). Figure 3 shows the HRs and 95% CIs for RDW, NLR, and their combined risk groups across the three Cox regression models.

### 3.4 Discriminative ability of the models

**Figure 4:**
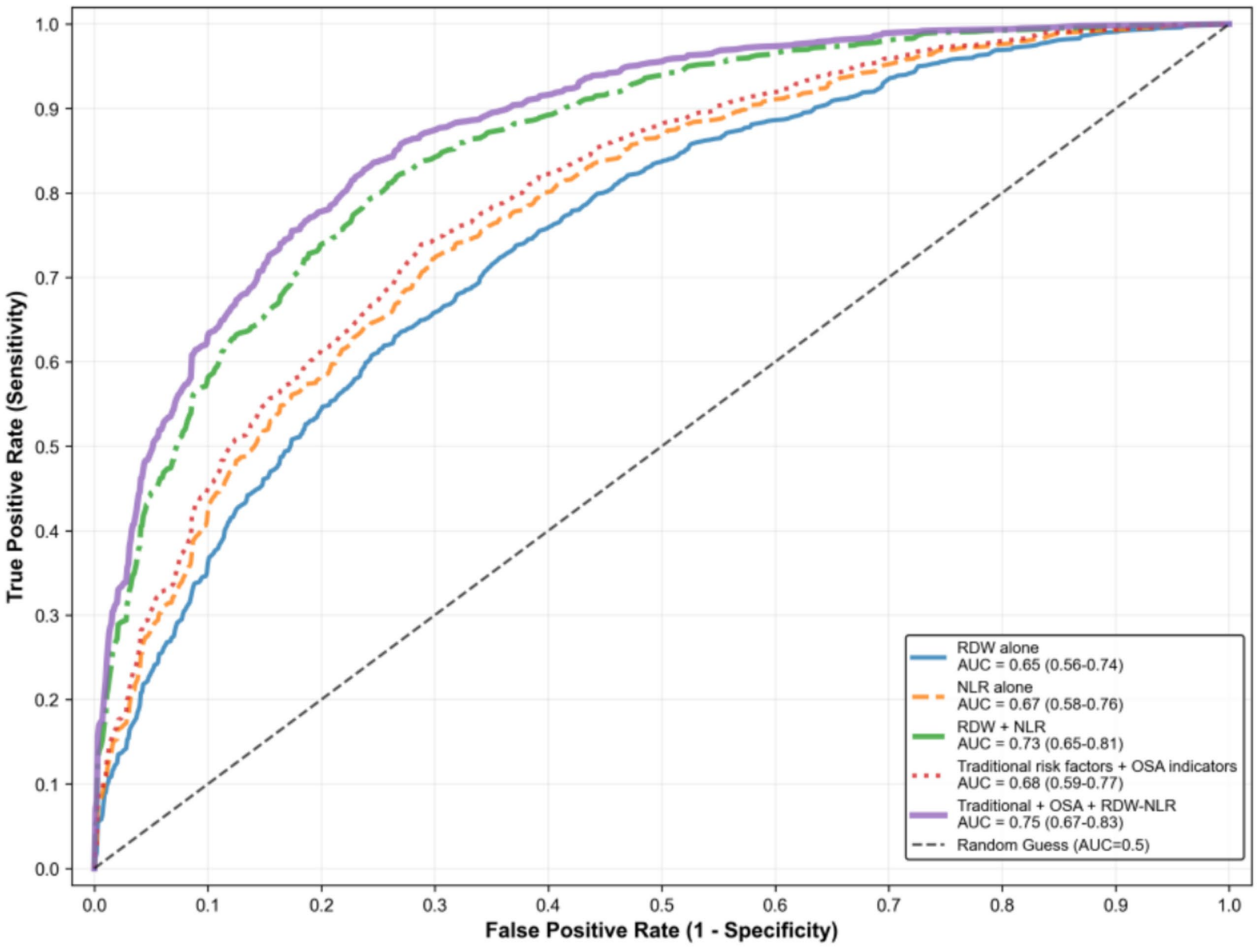
Discriminative ability of different models. AUC, area under the receiver operating characteristic curve; RDW, red blood cell distribution width; NLR, neutrophil-to-lymphocyte ratio; OSA, obstructive sleep apnea.

The discriminative ability of different models is summarized in Figure 4. The AUC of RDW alone was 0.65 (95%CI: 0.56–0.74), NLR alone was 0.67 (95%CI: 0.58–0.76), and the combined model was 0.73 (95%CI: 0.65–0.81), which was significantly higher than RDW alone (*p* = 0.031). The C-index of the combined model (0.72, 95%CI: 0.64–0.80) was higher than that of RDW alone (0.64) and NLR alone (0.66), with a lower AIC (198.6 vs. 206.3 and 203.5). Compared with the model containing traditional risk factors and OSA indicators (AUC = 0.68), adding the combined RDW-NLR index significantly improved reclassification (NRI = 0.42, 95%CI: 0.18–0.66, *p* = 0.001; IDI = 0.032, 95%CI: 0.008–0.056, *p* = 0.009).

### 3.5 Stratified and sensitivity analyses

**Figure 5:**
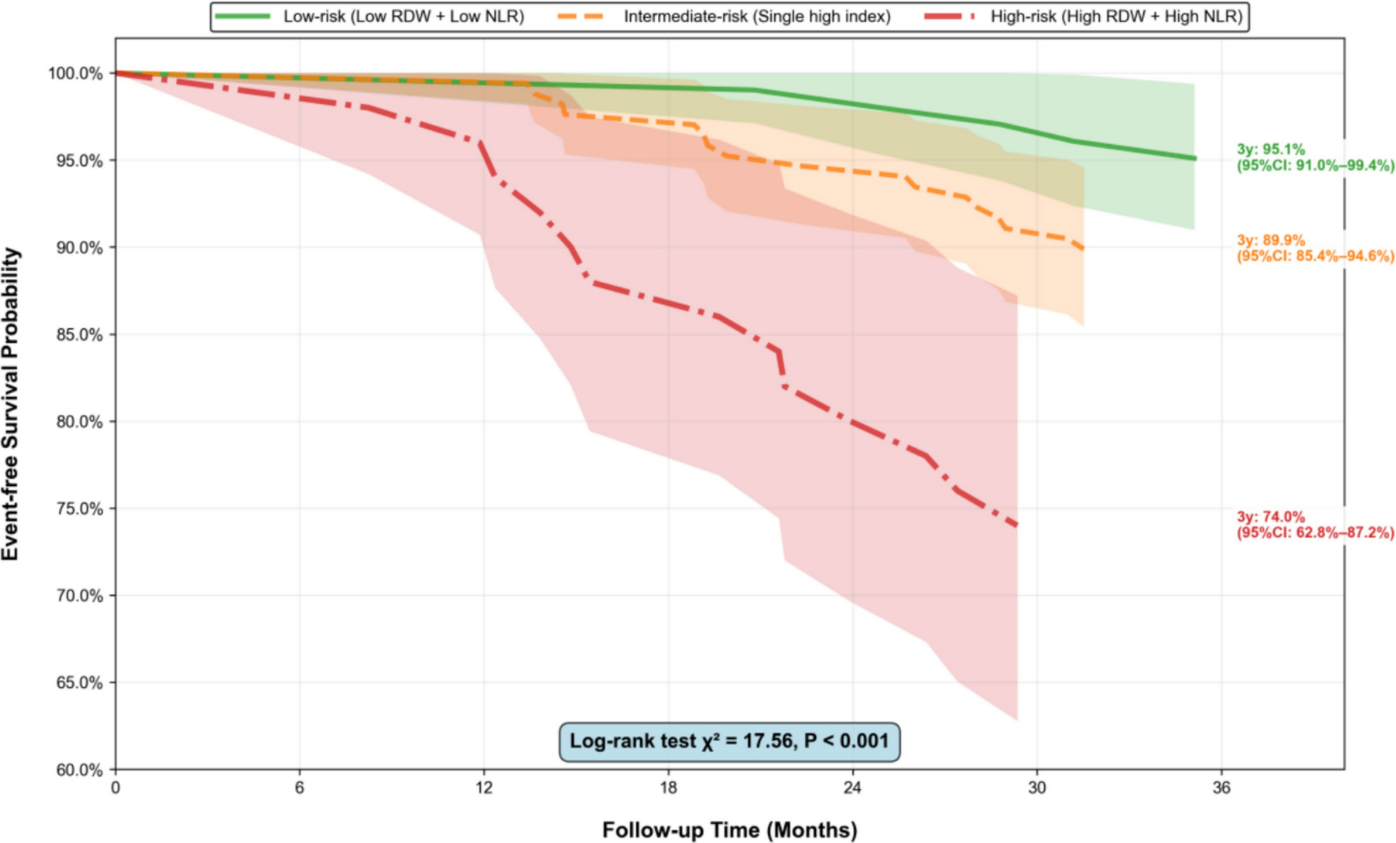
Kaplan–Meier survival curves by combined RDW-NLR risk groups. Kaplan–Meier curves for the cumulative incidence of composite cardiovascular events across three combined RDW-NLR risk groups (low-risk: low RDW + low NLR; intermediate-risk: single high index; high-risk: high RDW + high NLR). The Log-rank test *p* value is shown (*p* < 0.001), indicating a significant difference in event-free survival among the three groups. Numbers at risk are displayed below the *x*-axis (*n* = number of participants remaining at each time point). RDW, red blood cell distribution width; NLR, neutrophil-to-lymphocyte ratio.

**Figure 6:**
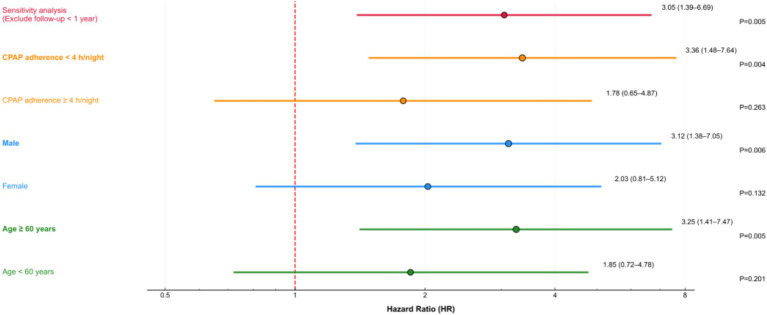
Forest plot of stratified analyses. HRs and 95% CIs for composite cardiovascular events in the high-risk group (vs. low-risk group) stratified by age (<60 years/≥60 years), gender, and CPAP adherence. All *p* values for interaction are >0.05, indicating no significant modification effect. CPAP, continuous positive airway pressure; HR, hazard ratio; CI, confidence interval.

The Kaplan–Meier survival curves (Figure 5) showed a significant difference in event-free survival across the three combined risk groups (Log-rank test *p* < 0.001), with the high-risk group having the lowest 3-year event-free survival rate. Stratified analysis showed that the association between the high-risk group and events was more pronounced in participants aged ≥60 years (HR = 3.25, 95%CI: 1.41–7.47, *p* = 0.005), males (HR = 3.12, 95%CI: 1.38–7.05, *p* = 0.006), and those with CPAP adherence <4 h/night (HR = 3.36, 95%CI: 1.48–7.64, *p* = 0.004), but no significant interaction was observed (all *p* for interaction >0.05;). Sensitivity analysis excluding participants with follow-up <1 year (*n* = 28) yielded consistent results (HR = 3.05, 95%CI: 1.39–6.69, *p* = 0.005 in Model 3). The proportional hazards assumption was satisfied for all exposure factors (all *p* for Schoenfeld residual test >0.05, Figure 6).

## 4 Discussion

This study explored the prognostic association of baseline RDW, NLR, and their combined index with 3-year composite cardiovascular events in patients with moderate-to-severe OSA through a prospective cohort design. The core findings showed that both RDW and NLR were independently associated with increased cardiovascular risk, and their combined risk stratification exhibited a significant dose–response relationship with outcomes. The combined RDW-NLR index demonstrated better discrimination compared to individual indicators, providing additional risk stratification value beyond traditional cardiovascular risk factors and OSA-specific parameters. These results confirm our initial hypothesis and offer new insights into cardiovascular risk stratification in this high-risk population.

The study verified that elevated baseline RDW and NLR were independently associated with cardiovascular events in moderate-to-severe OSA patients. Each 1% increase in RDW was associated with a 32% higher event risk, and each 1-unit increase in NLR corresponded to a 41% elevated risk. Rather than parallel biomarkers, RDW and NLR appear to reflect a unified pathophysiological process—the hypoxia-inflammation-erythropoiesis axis (17). In this framework, intermittent hypoxia raises hepatic hepcidin, restricting iron delivery to the bone marrow and widening red-cell volume heterogeneity, which manifests as elevated RDW (18). At the same time, hypoxia activates the NLRP3 inflammasome, prompting release of IL-1β and IL-18 that mobilize neutrophils and accelerate lymphocyte apoptosis, thereby increasing NLR (19). The convergence of these pathways promotes endothelial dysfunction and atherogenic priming, ultimately leading to composite cardiovascular events (20). By conceptualizing the two indices as components of this unified axis, we gain a testable model for future interventions aimed at either restoring iron availability or dampening inflammasome signaling. A previous study on acute myocardial infarction also confirmed that both RDW and NLR are robust predictors of short-term mortality, supporting the universal value of these two hematological indices in cardiovascular risk assessment (21).

The integration of multiple biomarkers and clinical parameters has emerged as a promising strategy to enhance cardiovascular risk prediction precision. Zhou et al. demonstrated that multimodal data integration significantly improved longitudinal prediction of cardiac and cerebrovascular events following initial OSA diagnosis, outperforming traditional single-parameter models (22). Similarly, Xu et al. developed a multimodal prognostic model for chronic coronary artery disease in non-OSA populations, achieving superior discrimination through comprehensive clinical data fusion (23). These studies establish that combining diverse data modalities can capture complex pathophysiological interactions more effectively than isolated markers. However, such sophisticated approaches often require advanced computational infrastructure, extensive data collection, and specialized expertise, creating barriers to widespread clinical implementation. Notably, combining RDW and NLR—two readily available hematologic markers—improves risk stratification for cardiovascular-related events in OSA patients compared to either index alone. This combination also adds incremental value to risk stratification when integrated into models that include traditional cardiovascular risk factors and polysomnographic severity indices (e.g., AHI) (24). This improvement reflects non-overlapping biology: NLR captures innate immune activation, while RDW mirrors erythropoietic stress (25, 26). Unlike earlier cohorts that reported similar NLR associations but ignored nocturnal hypoxic burden (SLT90 or ODI), we simultaneously adjusted for these parameters, yielding a more conservative estimate of residual inflammatory risk (3). Our prospective design also fills a methodological gap noted in recent meta-analyses: most prior studies were retrospective, omitted hypoxia indices, and failed to integrate hypoxic burden—an established predictor superior to AHI yet inconvenient to monitor routinely (27, 28). A CBC-derived RDW-NLR panel offers a low-cost, universally available alternative that summarizes both hematopoietic and inflammatory sequelae of intermittent hypoxia, providing primary-care-friendly refinement of cardiovascular risk in OSA.

The stratified analysis further revealed that the strength of the association for the combined high-risk group was more pronounced in patients aged ≥60 years, males, and those with poor CPAP adherence. Elderly OSA patients often have more comorbidities and a more severe chronic inflammatory state, which may amplify the pro-atherosclerotic effects of elevated RDW and NLR (29). For CPAP adherence, studies have shown that effective CPAP treatment can reduce NLR levels by alleviating intermittent hypoxia and chronic inflammation, which explains why patients with adherence <4 h/night had a higher event risk (HR = 3.36) (30, 31). This finding emphasizes the importance of improving CPAP adherence in high-risk OSA patients and suggests that dynamic monitoring of RDW and NLR may help evaluate treatment response.

For elderly male patients with poor CPAP adherence, more frequent follow-up and targeted adherence improvement strategies are needed to reduce cardiovascular risk. Additionally, the study results support the importance of integrating inflammatory and hematopoietic system biomarkers into OSA management guidelines. The combined RDW-NLR index, which indirectly reflects both hypoxic stress and inflammatory status, can complement existing assessment tools to improve the accuracy of risk stratification.

This study has several important limitations that should be considered when interpreting its findings. First, the single-center design conducted at one hospital in China may limit the generalizability of the results to other populations with different genetic backgrounds, healthcare settings, or lifestyles; multi-center validation is required to confirm the findings across diverse groups. Second, the availability of objective CPAP adherence data for only 58% of the participants introduces a potential for misclassification bias, and the impact of this incomplete data on the stratified analyses should be explicitly discussed. Furthermore, while the study hypothesizes that RDW reflects inflammatory erythropoietic stress, the absence of key iron metabolism metrics, such as ferritin or transferrin saturation, weakens the pathophysiological interpretation by making it difficult to rule out iron deficiency as a confounding factor. Lastly, the 3-year follow-up period may be insufficient to fully capture long-term cardiovascular risks associated with obstructive sleep apnea; longer-term data spanning 5 to 10 years would significantly strengthen the validity and clinical relevance of the findings.

## 5 Conclusion

In conclusion, the combined RDW-NLR index is a readily available prognostic marker for cardiovascular events in moderate-to-severe OSA patients. It reflects the synergistic effects of systemic inflammation and erythrocyte metabolic disorders induced by OSA, providing a new tool for clinical risk stratification and targeted intervention. This low-cost panel may facilitate risk stratification in resource-limited settings.

## Data Availability

The original contributions presented in the study are included in the article/Supplementary material, further inquiries can be directed to the corresponding author.
