# Supplementary material for: Combined red blood cell distribution width and neutrophil-to-lymphocyte ratio and risk of composite cardiovascular events in patients with moderate-to-severe obstructive sleep apnea: a prospective cohort study
Source: Front Med (Lausanne). 2026 Mar 11;13:1799058. doi: 10.3389/fmed.2026.1799058 (PMC13013362; doi:10.3389/fmed.2026.1799058)
Supplement: Supplementary file 1 [file Table_1.DOCX]

SUPPLEMENTARY FILE

Study Title: Combined Red Blood Cell Distribution Width and Neutrophil-to-Lymphocyte Ratio as a Predictor of Composite Cardiovascular Events in Patients with Moderate-to-Severe Obstructive Sleep Apnea: A Prospective Cohort Study

Study Site: Sleep Center, Xinxiang Central Hospital, Xinxiang City, Henan Province, China

Study Period: January 2020 - December 2022

IRB Approval: 2021-036-01[K]

---

INSTRUCTIONS: This questionnaire is to be completed by the study participant or study staff during the baseline enrollment visit. All information will be kept confidential and used for research purposes only.

---

PART A: DEMOGRAPHIC CHARACTERISTICS

A1. Study ID: ___________________

A2. Date of Enrollment: ____/____/________ (DD/MM/YYYY)

A3. Age: _______ years

A4. Gender: □ Male □ Female

A5. Ethnicity: _______________________________

A6. Height: _______ cm

A7. Weight: _______ kg

---

PART B: LIFESTYLE FACTORS

B1. SMOKING HISTORY

□ Never smoked (Skip to B2)

□ Former smoker (quit >1 year ago)

□ Former smoker (quit <1 year ago)

□ Current smoker

If former or current smoker:

- Age started smoking: _______ years

- If former smoker, age quit smoking: _______ years

- Average cigarettes per day: _______

- Pack-years: _______ (calculated as: packs/day × years smoked)

B2. ALCOHOL CONSUMPTION HISTORY

□ Never consumed alcohol

□ Former drinker (quit >1 year ago)

□ Former drinker (quit <1 year ago)

□ Current drinker

If current drinker:

- Average drinks per week: _______

- Type of alcohol usually consumed:

□ Beer □ Wine □ Spirits □ Mixed

B3. EXERCISE HABITS

□ Sedentary (no regular physical activity)

□ Light exercise (occasional, <3 times/week, <30 min/session)

□ Moderate exercise (3-5 times/week, 30-60 min/session)

□ Vigorous exercise (>5 times/week, >60 min/session)

---

PART C: CLINICAL HISTORY (Extracted from Electronic Medical Records)

C1. Hypertension: □ Yes □ No

If yes, duration: _______ years

Current medications: _______________________________

C2. Diabetes Mellitus: □ Yes □ No

If yes, type: □ Type 1 □ Type 2

Duration: _______ years

Current medications: _______________________________

C3. Dyslipidemia: □ Yes □ No

If yes, duration: _______ years

Current medications: _______________________________

C4. Other Comorbidities: _______________________________

---

PART D: MEDICATION USE

D1. Current medications (please list all):

_________________________________________________

_________________________________________________

_________________________________________________

---

PART E: POLYSOMNOGRAPHY PARAMETERS

E1. AHI (Apnea-Hypopnea Index): _______ events/hour

E2. ODI (Oxygen Desaturation Index): _______ events/hour

E3. Minimum SpO2: _______ %

E4. SLT90 (% time with SpO2 <90%): _______ %

---

PART F: LABORATORY INDICATORS

F1. RDW-CV (Red Blood Cell Distribution Width - Coefficient of Variation): _______ %

F2. Neutrophil Count: _______ ×10^9/L

F3. Lymphocyte Count: _______ ×10^9/L

F4. NLR (Neutrophil-to-Lymphocyte Ratio): _______ (calculated)

---

COMPLETED BY:

Staff Name: _________________________

Signature: _________________________

Date: ____/____/________

---

END OF QUESTIONNAIRE
